# The usefulness of ultrasound in predicting outcomes in patients with shoulder pain: a prospective observational study

**DOI:** 10.1093/rheumatology/kead546

**Published:** 2023-10-20

**Authors:** Gui Tran, Elizabeth M A Hensor, Sarah R Kingsbury, Philip O’Connor, Paul Cowling, Philip G Conaghan

**Affiliations:** Leeds Institute of Rheumatic and Musculoskeletal Medicine, University of Leeds, Leeds, UK; Department of Rheumatology, Harrogate and District NHS Foundation Trust, Harrogate, UK; Leeds Institute of Rheumatic and Musculoskeletal Medicine, University of Leeds, Leeds, UK; NIHR Leeds Biomedical Research Centre, Leeds, UK; Leeds Institute of Rheumatic and Musculoskeletal Medicine, University of Leeds, Leeds, UK; NIHR Leeds Biomedical Research Centre, Leeds, UK; Leeds Teaching Hospitals NHS Trust, Leeds, UK; Leeds Institute of Rheumatic and Musculoskeletal Medicine, University of Leeds, Leeds, UK; NIHR Leeds Biomedical Research Centre, Leeds, UK

**Keywords:** shoulder pain, ultrasound, prognosis, diagnostic imaging, organization of health care

## Abstract

**Objectives:**

Shoulder pain is common but current clinical classification has limited utility. We aimed to determine whether groups of ultrasound-based shoulder pathologies exist and to evaluate outcomes according to identified groups and individual pathologies.

**Methods:**

This was a prospective study of a community-based cohort with shoulder pain referred for their first ultrasound scan at a single radiology unit, with subsequent routine clinical care. Patient-reported outcomes were collected at baseline, 2 weeks and 6 months; standardized ultrasound reporting was employed. Latent class analysis (LCA) identified ultrasound pathology–based groups. Multiple linear regression analysis explored associations between baseline pathologies, subsequent treatment and Shoulder Pain and Disability Index (SPADI). Short-term response to corticosteroid injections was investigated.

**Results:**

Of 500 participants (mean age 53.6 years; 52% female), 330 completed follow-up. LCA identified four groups: bursitis with (33%) or without (27%) acromioclavicular joint degeneration, rotator cuff tear (21%) and no bursitis/tear (19%). Total SPADI was higher at baseline for cuff tears (mean 55.1 *vs* 49.7–51.3; overall *P* = 0.005), but accounting for this, groups did not differ at 6 months (43.5 *vs* 38.5–40.5; *P* = 0.379). Baseline SPADI was the only predictor of 6-month SPADI retained by penalized modelling; neither LCA-derived ultrasound groups nor individual pathologies were selected. Response to baseline injection at week 2 did not differ between groups (mean SPADI 40.1–43.8; *P* = 0.423).

**Conclusion:**

Ultrasound-based classification (groups or individual pathologies) of shoulder pain did not predict medium-term outcomes using current treatments. The role of routine diagnostic ultrasound for shoulder pain needs consideration; it may be useful to establish evidence-based therapies for specific pathologies.

Rheumatology key messages

- Four different patterns of ultrasound-detected pathologies were confirmed.
- Ultrasound findings did not predict differences in short/medium-term outcomes in a usual care pathway.
- Ultrasound in shoulder pathways need re-evaluation and may only be useful for established evidence-based treatments.

## Introduction

Shoulder pain is one of the most common musculoskeletal complaints [1, 2] and 50% remain symptomatic after 18 months [3]. The socioeconomic burden is large, with one Swedish study estimating mean annual costs to be €4139/patient [4]. Healthcare utilization is substantial: it accounts for 1% of UK general practitioner consultations, and 5% of patients are referred to secondary care in the USA [5].

Treatment of shoulder pain is limited, and identification of subgroups is required to better understand responses [6]. Clinical classification has poor reliability and the utility of clinical examination for discriminating shoulder pathologies is uncertain [7]. To help with diagnosis and management, ultrasound (US) is increasingly used [8]. However, the extent to which imaging informs management and improves outcomes is uncertain [9], and US for shoulder pain is not routinely recommended [10]. Few studies have explored the association of US-detected pathologies with patient outcomes [11]. The importance of rational diagnostic testing to improve care and reduce costs has been highlighted [12].

A retrospective study identified groups with different patterns of US pathologies [13]. We hypothesized that accurate US-based classification would predict patient outcomes and short-term response to steroid injection. The current prospective study aimed to confirm the existence of US-based diagnostic groups and determine whether US-detected pathology predicted medium-term outcomes in the context of usual care. Short-term response to IA corticosteroid (CS) injections, used for their anti-inflammatory effects, was also evaluated.

## Methods

### Study design and source population

This prospective, observational study recruited 500 community-based patients referred from primary care for US of a painful shoulder from October 2016 to December 2017. Scans were performed in a single radiology unit in England.

Inclusion criteria were patients referred from primary or intermediary care with shoulder pain, aged >18 years, with ability to provide informed consent and undergoing first shoulder US. Exclusion criteria were inflammatory arthritis, previous shoulder trauma/surgery, complex regional pain syndrome, CS injection/physiotherapy in the prior 6 weeks or referral for reasons other than shoulder pain (e.g. soft tissue mass). This was excluded by individually reviewing free texts on the referral. Referrals could be through any means consistent with the usual local care pathway, including community musculoskeletal physiotherapists.

Patients received usual care post-scan, including US-guided CS injections according to general practitioner request and US findings.

The study protocol was approved by the North East—Newcastle & North Tyneside 2 Research Ethics Committee (16/NE/0108) and given Research and Development permission from Leeds Teaching Hospitals NHS Trust [R&I number RR16/128 (201260)]. Participants gave written informed consent before taking part.

### Data collection

Patients completed paper questionnaires at baseline (clinic visit) and 6 months (via mail) recording age, gender, BMI, pain duration prior to scan, current pain (yes/no), comorbidities and other joints affected. Outcome measures included the Shoulder Pain and Disability Index (SPADI) [14], Oxford Shoulder Score (OSS) [15], Pain-DETECT [16], quality of life (EQ5D-5L) [17], Hospital Anxiety and Depression Scale (HADS) [18], Brief Illness Perception Questionnaire (Brief IPQ) [19], Pain Self-Efficacy Questionnaire (PSEQ) [20] and Brophy & Marx shoulder activity scale [21]. Guidelines for shoulder problems recommend treatment with physiotherapy, CS injections, analgesics or surgery [22]. Patients reported prior treatments at baseline 3 and 6 months. To verify analgesia use, repeat prescriptions for analgesia were identified from primary-care electronic records [23].

To assess short-term response to CS injections, patients also completed SPADI by e-mail or telephone at 2 weeks (based on likely optimal efficacy).

Patients with no US-detected pathology were invited for clinical assessment within 4 weeks.

During a consensus session, the Radiology Department agreed definitions, standardized scanning [24] and reporting for the 10 most common US pathologies (reported as present/absent) identified from previous work [13] (Supplementary Table S1, available at *Rheumatology* online). Three consultant radiologists, two specialist trainees and three senior sonographers undertook scanning using GE LOGIQ E9 machines. Image acquisition and reporting occurred concurrently.

To avoid recruitment bias, quota sampling was used in four categories: gender (male/female) and age (younger/older split at the median), based on proportions from a previous study [13].

### Statistical analysis

Our primary outcome was SPADI at 6 months. Latent class analysis (LCA) identified US-based groups [25]. Regression analyses compared outcomes between groups.

All questionnaires except EQ5D-5L were assessed for fit to the Rasch model [26].

A preparatory Little’s test indicated no departure from missing completely at random (*P* = 1.000) [27]. To address missing covariate data, multiple imputation by chained equations imputed 20 complete datasets [28, 29]; results were combined according to Rubin’s rules [30].

Baseline characteristics were compared between follow-up questionnaire responders and non-responders, to check for responder bias.

Each scanned pathology was included in the LCA model; partial and full rotator cuff (RC) tears were combined, i.e. RC tear absent/present. Optimum group number was identified from models without covariates, with 1000 random starts, using the Bayesian Information Criterion (BIC). Bootstrapped likelihood ratio tests (BLRT) also compared fit between nested solutions; additional model fit statistics were calculated. Final LCA groupings were identified following multiple imputation, with covariates included. The pathologies included in the LCA were: RC tear; bursal thickening; dynamic subacromial impingement; calcific tendinitis; acromioclavicular joint (ACJ) pathology; glenohumeral OA; adhesive capsulitis; biceps tenosynovitis; and RC tendinopathy. The covariates were age; sex; symptom duration; physiotherapy before scan; number of steroid injections received prior to scan; post-baseline injections, physiotherapy and surgery received (each yes/no); whether the participant habitually used their arms to rise from a chair; steroid injection given at time of scan; EQ5D health index and visual analogue scale; height; weight; number of painful joints; baseline activity score, P-SEQ, brief IPQ, Oxford shoulder score, HADS and painDetect; SPADI at baseline, 6 and 12 months; and patient-acceptable symptom state (PASS) at baseline and 6 months.

A classify>predict approach was adopted, using posterior probabilities of group membership obtained following multiple imputation (Supplementary Material, available at *Rheumatology* online).

#### Comparisons between pathology groups identified via LCA

Baseline characteristics and post-baseline treatments were compared between groups using linear, quantile or logistic regression (binary or ordinal), according to the outcome type, including age, sex and symptom duration as covariates.

To predict 2-week and 6-month SPADI, multivariable linear regression analysis included the following baseline covariates, selected *a priori*: pathology group, steroid injection at scan, age, sex, pain duration prior to scan, use arms to rise from chair (yes/no), prior physiotherapy (yes/no), number of previous steroid injections, activity score, P-SEQ, brief IPQ, HADS and painDetect. All variables were forced into the multivariable ordinary least squares regression model; penalized regression was then used to identify a more parsimonious model (see Supplementary Methods, available at *Rheumatology* online).

In a sensitivity analysis, groups with similar trajectories of total SPADI over time were identified using growth mixture modelling (see Supplementary Methods, available at *Rheumatology* online). Trajectory classes were compared descriptively between US pathology groups and according to whether a steroid injection was received at scan.

All analyses were conducted at the two-sided 5% level of significance, except interactions, tested at the two-sided 10% level. Appropriate checks were made that the data satisfied test assumptions. Analyses used Stata v15.0 and the LCA plugin v1.2f [25], and R v4.0.2 [31].

#### Sample size

To accurately identify the number of groups using BIC required *n* = 500 as LCA included <10 categorical outcomes with an assumed unbalanced, complex structure [32].

Rules of thumb for linear regression analysis require 50 + 8m patients (m = independent variables) [33]. Analysis from our retrospective study suggested four or five groups [13], generating up to four dummy variables; adding 17 covariates, this required 218 patients. However, there was the potential for over-fitting. Using the PEAR technique, to maximize precision efficacy in future samples, assuming R^2^ = 0.40 and requiring precision efficacy to be ≥75% this required a sample size of 286 patients. We recruited 500 patients, allowing <40% drop-out, as previously reported for surveys [34].

## Results

We recruited 500 patients (52% women, mean age 53.6 years); 496 had SPADI data at baseline, 384 at 2 weeks and 330 at 6 months. Patients had a median of three US-detected pathologies (Table 1).

**Table 1.** Baseline characteristics of patients recruited, by questionnaire completion status (baseline and 6 months, baseline only)

	All patients (*n* = 500)	Completed at baseline and 6 months (*n* = 330)	Completed only at baseline (*n* = 170)
Age, years, mean (s.d.)	53.6 (14.5)	57.4 (13.5)	46.2 (13.5)
Female, % (*n*)	52 (258)	55 (182)	45 (76)
Duration of symptoms, months, median (IQR)	5 (3, 10), *n* = 361	6 (3, 10), *n* = 242	5 (3, 10), *n* = 119
RC tear (y/n), % (*n*)	25 (125)	29 (99)	16 (28)
Full thickness RC tear, % (*n*)	17 (87)	21 (69)	11 (18)
Bursitis, % (*n*)	71 (354)	71 (234)	71 (120)
Impingement, % (*n*)	59 (297)	63 (208)	52 (89)
Calcific tendinitis, % (*n*)	8 (41)	9 (29)	7 (12)
ACJ degeneration, % (*n*)	47 (235)	52 (173)	36 (62)
Glenohumeral OA, % (*n*)	3 (17)	4 (13)	2 (4)
Adhesive capsulitis, % (*n*)	8 (39)	9 (28)	6 (10)
Biceps tenosynovitis, % (*n*)	4 (22)	4 (14)	5 (8)
RC tendinopathy, % (*n*)	29 (147)	32 (107)	24 (40)
US pathology absent, % (*n*)	7 (34)	4 (13)	12 (21)
Number of pathologies, median (IQR)	3 (2,3)	3 (2, 4)	2 (1, 3)
BMI, kg/m^2^, mean (s.d.)	27.6 (5.1) *n* = 461	27.9 (5.1), *n* = 314	26.9 (5.0), *n* = 147
Number of painful joints, median (IQR)	3 (1–5)	3 (1–5)	2 (1–5)
HADS baseline total, mean (s.d.)	14.0 (5.9), *n* = 490	13.3 (6.0), *n* = 324	15.4 (5.6), *n* = 166
SPADI baseline total, mean (s.d.)	51.5 (9.8), *n* = 496	51.2 (9.7), *n* = 328	50.5 (13.3), *n* = 167

ACJ: acromioclavicular joint; HADS: Hospital Anxiety and Depression Scale; IPQ: Illness Perception Questionnaire; IQR: interquartile range; PASS: patient-acceptable symptom state; P-SEQ: Pain Self-Efficacy Questionnaire; RC: rotator cuff; SPADI: Shoulder Pain and Disability Index.

All scales fit the Rasch model (Supplementary Table S2, available at *Rheumatology* online). Total SPADI showed no evidence of multidimensionality; sensitivity analysis (not shown) using SPADI pain subscale instead of total SPADI did not alter findings.

Table 1 reports baseline characteristics of 6-month questionnaire completers and non-completers. Non-completers were younger, more anxious and depressed, and likely to have no pathologies on US. Baseline pain and disability was similar.

### Identifying groups using LCA

The *a priori*–selected criterion BIC was similar for three- and four-group solutions. BLRT (demonstrated to be the best performing of the available criteria [35]) favoured four groups over three (*P* = 0.010); additional fit statistics were split as to the best model (Supplementary Table S3, available at *Rheumatology* online). We retained a four-group solution as this had clinical face-validity. The groups showed similar patterns of pathologies compared with those identified in earlier work [13] (Supplementary Table S4, available at *Rheumatology* online). We interpreted what each group broadly represented clinically according to the most/least prevalent pathologies, and then checked the accuracy of these characterizations. Patients could be accurately assigned to the correct group in 92% of cases using ‘bursitis without ACJ degeneration or RC tear’ (group 1); ‘bursitis with ACJ degeneration but no RC tear’ (group 2); ‘RC tear with/without bursitis’ (group 3); and ‘no bursitis or RC tear’ (group 4). However, the LCA-derived groups were used for subsequent analysis.

### Baseline characteristics of pathology groups

At baseline, 34/500 patients had no US-detectable pathology, constituting 36% of group 4 (no bursitis or RC tear; Table 2). Those with pathologies in this group tended to have just one or two co-occurring pathologies (Supplementary Fig. S1, available at *Rheumatology* online), compared with median two to four in the other groups.

**Table 2.** Summary of baseline characteristics of pathology groups (imputed data; all patients)

	Group 1: bursitis w/o ACJ degeneration or RC tear	Group 2: bursitis with ACJ degeneration, w/o RC tear	Group 3: RC tear	Group 4: no bursitis or RC tear	Overall *P-*value^a^
% of sample	33	27	21	19	
Age, years, mean (95% CI)	47.0 (44.8, 49.2)	55.5 (53.1, 57.8)	65.1 (62.3, 67.9)	49.5 (46.8, 52.1)	
Female, %	56	52	45	51	
Duration, months, median (95% CI)	5.2 (3.0, 9.9)	5.6 (3.0, 9.9)	4.8 (2.9, 9.6)	5.9 (3.7, 10.8)	
RC tear (y/n), %	3	5	>99	2	
Full thickness RC tear, %	<1	<1	83	<1	
Bursitis, %	>99	98	49	4	
Impingement, %	65	69	89	4	
Calcific tendinitis, %	9	11	2	12	
ACJ degeneration, %	<1	98	64	36	
Glenohumeral OA, %	2	<1	12	2	
Adhesive capsulitis, %	3	5	6	22	
Biceps tenosynovitis, %	<1	5	13	2	
RC tendinopathy, %	18	45	29	18	
Probability of membership, mean	0.89	0.94	0.93	0.96	
US pathology absent, %	<1	<1	<1	36	
Number of pathologies, median (95% CI)	2.0 (2.0, 2.6)	3.0 (3.0, 4.0)	3.8 (3.0, 4.1)	1.0 (0.0, 2.0)	*P* < 0.001
Injection at time of scan, %	59	49	10	16	*P* < 0.001
BMI, mean (95% CI)	27.4 (26.5, 28.3)	28.5 (27.6, 29.5)	27.1 (26.0, 28.2)	27.3 (26.2, 28.5)	*P* = 0.201
Number of painful joints, median (95% CI)	2.4 (1.0, 4.0)	3.0 (1.9, 5.1)	2.0 (1.0, 4.0)	2.6 (1.0, 4.7)	*P* = 0.159
Uses arms to rise from chair, %	37	53	56	49	*P* = 0.388
Physiotherapy before baseline, %	26	25	28	32	*P* = 0.670
Number of injections, %					*P* = 0.867
1	17	15	18	11	
2	5	7	5	7	
EQ5D-5L index, median (95% CI)	0.80 (0.69, 0.87)	0.77 (0.61, 0.86)	0.72 (0.53, 0.84)	0.80 (0.68, 0.87)	*P* = 0.712
EQ5D-5L VAS, median (95% CI)	72.3 (60.0, 85.0)	71.3 (50.0, 85.0)	78.3 (64.0, 89.5)	75.0 (60.0, 85.0)	*P* = 0.325
Shoulder activity score, mean (95% CI)	10.6 (10.1, 11.2)	9.6 (8.8, 10.4)	9.5 (8.6, 10.4)	9.8 (9.0, 10.5)	*P* = 0.290
Total SPADI, mean (95% CI)	50.3 (48.8, 51.8)	51.3 (49.7, 53.0)	55.1 (53.0, 57.2)	49.7 (47.7, 51.7)	*P* = 0.005
Oxford shoulder, mean (95% CI)	33.9 (33.0, 34.9)	34.1 (33.0, 35.2)	36.6 (35.3, 37.9)	34.0 (32.6, 35.3)	*P* = 0.091
P-SEQ, mean (95% CI)	39.3 (37.7, 40.9)	37.7 (35.9, 39.5)	37.7 (35.7, 39.7)	39.5 (37.4, 41.6)	*P* = 0.085
Brief IPQ, mean (95% CI)	42.4 (41.4, 43.5)	42.1 (40.9, 43.4)	43.1 (41.7, 44.5)	42.3 (40.9, 43.7)	*P* = 0.162
HADS, mean (95% CI)	13.8 (12.9, 14.8)	14.1 (13.1, 15.2)	14.6 (13.4, 15.8)	13.5 (12.3, 14.7)	*P* = 0.160
painDetect, mean (95% CI)	14.3 (13.4, 15.2)	13.9 (12.9, 14.9)	14.7 (13.6, 15.8)	13.4 (12.3, 14.6)	*P* = 0.193
PASS at baseline, %	14	23	18	36	*P* = 0.002

a Overall *P*-value for US pathology group, adjusted for age, sex and symptom duration. ACJ: acromioclavicular joint; HADS: Hospital Anxiety and Depression Scale; IPQ: Illness Perception Questionnaire; PASS: patient-acceptable symptom state; P-SEQ: Pain Self-Efficacy Questionnaire; RC: rotator cuff; SPADI: Shoulder Pain and Disability Index.

Adjusting for age, sex and symptom duration, baseline SPADI was highest in group 3 (RC tear) and lowest in group 4; however, differences were small. In patients with bursitis (*n* = 354), 55% (155/283) without RC tears received injection at their scan compared with 21% (15/71) with RC tears; this reflected differing rates of injection at time of scan between groups 1 and 2, and group 3, despite large proportions of patients with bursitis in these groups.

Proportions of patients receiving injections at their scan by individual pathologies are presented in Supplementary Table S5 (available at *Rheumatology* online).

### Post-baseline treatment

Medication records were accessed after follow-up in 313/330 patients with 6-month SPADI available. We found no differences between groups in repeat prescriptions for opioids or NSAIDs (Supplementary Table S6, available at *Rheumatology* online). Adjusting for age, sex and symptom duration, estimated proportions for groups 1–4 were: opioids 12%, 18%, 9% and 9% (overall *P* = 0.289), and NSAIDs 6%, 13%, 8% and 6% (overall *P* = 0.347).

Combining results from the 3-month and 6-month follow-up, data on post-baseline physiotherapy, injections and surgery were available for 296, 285 and 299 patients, respectively, of the 330 with 6-month SPADI available. Using imputed data for post-baseline treatments, there were no differences between groups in patients reporting receiving physiotherapy (55%, 53%, 67% and 58%; overall *P* = 0.476), but those in groups 1 and 2 (bursitis predominant) were more likely to report injections (61%, 56%, 35% and 38%; overall *P* = 0.018).

We confirmed surgery during follow-up in 18 patients. However, three did not report having surgery, whilst 22 different patients reported surgery which we could not confirm, perhaps reflecting private treatment. More of those with RC tears (group 3) received surgery. Adjusted proportions reporting surgery were 11%, 5%, 26% and 10% (overall *P* = 0.011) in groups 1–4, respectively.

### Association between group membership and symptoms at 6 months

There were 330 patients with 6-month SPADI data. Adjusting for age, sex and HADS there was no difference between groups in 6-month questionnaire return rate (63%, 70%, 69% and 64% in groups 1–4, respectively; *P* = 0.601).

At 6 months median (interquartile range; range) total SPADI was 45 (32, 52; 0–78) and 76% (251/330) still scored ≥30/100 for total SPADI using Rasch model–derived interval-scaled scores. Mean change from baseline (95% CI) was –11.0 (–12.9, –9.2), within minimum detectable change (18 units [36]). Patient symptom state was still unacceptable at 6 months in 37% (119/320 with data). In a multivariable model, pathology group did not predict SPADI score at 6 months (overall *P* = 0.379; Table 3). R^2^ was low (R^2^ 0.28; adjusted R^2^ 0.24). Sensitivity analysis using individual pathologies instead of LCA groups did not affect these conclusions (Supplementary Table S7, available at *Rheumatology* online). Penalized regression retained only baseline SPADI as a predictor, irrespective of whether LCA-derived US groups or individual pathologies were included in the initial predictor list.

**Table 3.** Predictors of total SPADI score at 6 months

Baseline characteristic	Multiple linear regression
	Coefficient^a^ (95% CI), *P*-value
Pathology group	
Bursitis w/o ACJ degeneration	Reference
Bursitis with ACJ degeneration	0.08 (–5.15, 5.32), *P* = 0.975
RC tear	5.01 (–1.48, 11.50), *P* = 0.130
No bursitis, no RC tear	1.98 (–4.00, 7.96), *P* = 0.516
Injection at time of scan	4.87 (0.40, 9.34), *P* = 0.033
Age, years	–0.01 (–0.17, 0.15), *P* = 0.898
Female	–1.51 (–5.42, 2.40), *P* = 0.448
Duration of symptoms, months	0.00 (–0.04, 0.05), *P* = 0.879
Uses arms to rise from chair	2.65 (–1.50, 6.81), *P* = 0.210
Had physiotherapy before baseline	–0.23 (–4.78, 4.31), *P* = 0.920
Had 1 injection before baseline	2.35 (–2.87, 7.56), *P* = 0.377
Had ≥2 injections before baseline	6.53 (–2.50, 15.57), *P* = 0.156
Total SPADI score	0.62 (0.35, 0.89), *P* < 0.001
Shoulder activity score	–0.59 (–1.15, –0.04), *P* = 0.037
P-SEQ score	–0.20 (–0.46, 0.07), *P* = 0.141
Brief IPQ score	0.19 (–0.16, 0.54), *P* = 0.298
HADS score	0.01 (–0.38, 0.41), *P* = 0.947
painDetect score	–0.05 (–0.46, 0.35), *P* = 0.790
Constant^b^	35.44 (29.50, 41.38), *P* < 0.001
R^2^; adjusted R^2^; root mean squared error	0.28; 0.24; 15.96

a Interpreted as unit difference in Rasch-transformed SPADI score per 1 additional unit of the independent variable.

b Estimated total SPADI at 26 weeks in patients in the reference category for all categorical variables and with mean values for continuous covariates. ACJ: acromioclavicular joint; HADS: Hospital Anxiety and Depression Scale; IPQ: Illness Perception Questionnaire; P-SEQ: Pain Self-Efficacy Questionnaire; RC: rotator cuff; SPADI: Shoulder Pain and Disability Index; w/o: without.

### Short-term differences in symptoms following baseline steroid injection

Of 384 participants who provided SPADI data at week 2, 150 (39%) had received baseline steroid injections, compared with 40/116 (34%) without 2-week SPADI.

In the 384 patients with 2-week data, proportions receiving baseline injections were 58%, 51%, 11% and 18% in groups 1–4, respectively. The extent of the difference at 2 weeks predicted by baseline injection did not differ between US groups (interaction group × injection, *P* = 0.354). Having removed the interaction, 2-week SPADI did not differ by US group [difference compared with group 1 was –1.56 (–6.16, 3.05), –2.14 (–6.71, 2.43) and –3.63 (**–**7.45, 0.20) for groups 2–4, respectively; overall *P* = 0.423]. The estimated difference between steroid-treated and untreated patients was –11.0 (95% CI –13.8, –8.2), which although statistically significant (*P* < 0.001) did not exceed SDC.

### Trajectories of change in SPADI over 6 months

**Figure 1.**
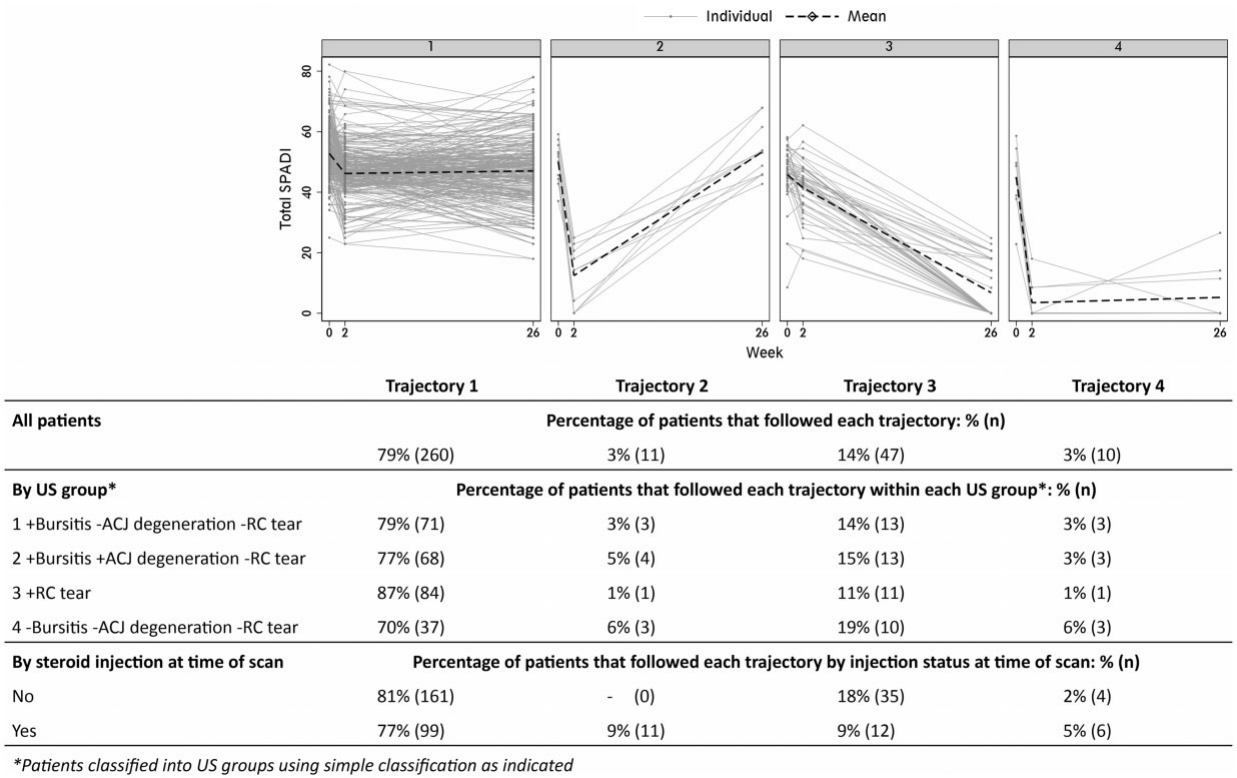
Trajectories of change in total SPADI

Four trajectory classes were identified in *n* = 228 patients (Fig. 1; Supplementary Results; Supplementary Table S8, available at *Rheumatology* online). The majority (79%; 260/328) showed little change over time; estimated mean SPADI in this class was 52.8 at baseline and 47.1 at week 26 (trajectory 1); nevertheless, 57% reported achieving a PASS at week 26 compared with just 18% at baseline. Eleven patients, all of whom had received a baseline steroid injection, improved rapidly at week 2 but had returned to baseline symptoms by 6 months (trajectory 2); only 2 (18%) achieved PASS at week 26. A minority of patients (17%) showed either a gradual [14% (47/328); trajectory 3] or rapid sustained improvement [3% (10/328); trajectory 4]; almost all achieved PASS at week 26 (95% and 100%, respectively).

The majority of patients followed trajectories 1 or 2, with little change relative to baseline at week 26, irrespective of US pathology group (groups 1–4: 82%, 82%, 88% and 76%).

### Patients with no US-detectable pathology

Mean baseline SPADI did not differ between patients without US-detectable pathology at baseline (*n* = 34) and those with any pathology [*n* = 462; difference (95% CI) unadjusted 2.10 (–1.31, 5.51), *P* = 0.227; adjusted for age, sex and symptom duration –0.12 (–3.56, 3.32), *P* = 0.945]. Eight attended for review: one reported resolving symptoms; two adhesive capsulitis; two neck pathologies with referral to shoulder; two scapula disorders; and one uncertain diagnosis. Twenty-six did not attend: 18 did not respond/declined involvement; 8 reported improved/resolved symptoms.

## Discussion

To our knowledge this is the largest prospective, longitudinal study on community-based patients undergoing an US of their painful shoulder, and the first to investigate groups with distinct patterns of US pathologies in predicting outcome.

This study demonstrated that US-detected pathologies grouped or individually, do not predict medium-term outcomes in a usual care pathway.

LCA was used to identify whether subgroups of US pathologies existed when used in a usual care pathway and *a priori* clinical input had already been provided to request the scan. Four groups with different patterns of US-detected shoulder pathologies were found: bursitis with no ACJ degeneration; bursitis with ACJ degeneration; RC tears; and no RC tear or bursitis. These groups differed only slightly from our earlier, larger (*n* = 3000) retrospective study [13]. In the current study, we included more covariates in the LCA model, improving classification accuracy. Given the overlap with our earlier study, which had the power to detect a larger number of groups had they existed, we believe we have not unduly simplified the data by classifying the participants agnostically into four groups, as opposed to examining each pathology individually or using *a priori* clinician-defined groups.

These four groups may represent a chronological progression of shoulder problems, as the ‘bursitis without ACJ degeneration’ group and group with ‘no tears or bursitis’ were youngest. Patients with ‘bursitis with ACJ degeneration’ were the next oldest, followed by ‘RC tear’. ACJ degeneration increases with age, and if we consider that ACJ degeneration may be an incidental finding (radiographic OA can be asymptomatic [37]), three groups exist: bursitis without tears, tears (with or without bursitis) or neither of these pathologies. These pathologies help define the groups but do not fully describe them. Of note, half of those with RC tears also had bursitis, and the majority of patients with bursitis and/or RC tears also had impingement. Many patients without bursitis or RC tear had calcific tendinitis, adhesive capsulitis or tendinopathy. Whilst adhesive capsulitis is a clinical diagnosis, US features are seen, and dynamic US offers the opportunity for both clinical and radiological examination.

Short-term improvements following steroid injections at baseline were within MCID. We found no differences in short-term response to steroids between groups.

Although those in the bursitis groups were more likely to receive injection and those in the RC tear group were more likely to receive surgery after scan, we found no differences in 6-month outcomes between groups. This suggests that although US diagnosis may influence treatment received, it does not predict medium-term outcome.

Investigation of patient trajectories revealed that, although some patients improved, the majority showed minimal change over time. Similar trajectories have been shown in knee OA [38], and further studies are required to identify the patient characteristics resulting in these different trajectories.

Pathologies exist in symptomatic and asymptomatic shoulders [39] and the relationship between imaging-detected pathology and symptoms is poorly understood. We did not assess the structure–pain relationship as our population only included patients with shoulder pain and did not capture the full range of covariates necessary for this analysis.

There were limitations to this study. Although 500 patients provided baseline data for our primary outcome, only 330 (66%) completed follow-up, which may result in bias. However, this is considered an acceptable response rate for a survey, as the overall response rates for surveys across 1607 studies was 48.3% [34]. Although those who completed follow-up were older and less anxious and depressed than non-completers, other characteristics including SPADI were similar. This study was observational and there may be treatment recall bias. This study evaluated outcomes according to current usual care pathways, therefore treatment, including physiotherapy and steroids, may have varied in dose, frequency and duration and compliance. There may be channelling bias to treatment by pathologies, although we adjusted for reported pre-baseline treatment. We could not include over-the-counter NSAIDs. Other than full and partial tears, the severity of US pathologies was not assessed and severity, rather than presence of pathology, may be important predictors of outcome. We had one US time-point, at baseline. It would be interesting to see how pathologies change over time, whether the described groupings change and whether patients move between groups. A recent review found enlarging RC tears was associated with increased incidence of symptoms, although combined pathologies was not assessed [11]. Although previous work has shown that the inter-rater reliability for two of the present sonographers was acceptable [40], we did not explore the inter/intra-reader reliability of all sonographers. When assessing patients with no US pathology, the clinician was aware of the US results. We were unable to access primary/community care records or neighbouring hospital records to capture surgical events, and were reliant on patient recall/our Trust records for surgical events.

The role of US scans in improving the care of shoulder pain requires further evaluation. A recent randomized control trial found no difference at 12 months on patient-perceived recovery in those receiving individual US pathology tailored treatment compared with usual care, although combination of pathologies was not assessed and the study was under-enrolled by 50% [41]. A recent study comparing subacromial decompression *vs* arthroscopy alone or conservative treatment for subacromial pain [42], diagnosed using imaging including US, found no clinically significant differences between groups in the medium- to long-term. European guidelines advise that US of the shoulder should be used as the first choice imaging technique for bursitis, RC tears, calcific tendonitis and long head of the biceps (LHB) pathologies [43]. However, these guidelines were developed from expert opinion using available evidence, and did not evaluate the predictive value of US pathologies. The findings from this shoulder pain study are similar to studies on back pain. Research has shown that routine imaging does not affect 12-month outcomes in patients receiving usual care for lower back pain without features suggesting serious underlying conditions [44].

In conclusion, medium-term outcomes of patients undergoing their first shoulder US did not differ by pathology group membership or individual pathologies. Given our finding that the use of US does not predict medium-term outcomes in a usual care situation, and if symptoms tend to stay stable or improve to the same extent irrespective of the pathology present, knowing which specific pathologies are present may only be useful if effective, evidence-based treatments for specific (groups of) pathologies are established. The precise role of US in the shoulder pain pathway therefore remains uncertain and further work is required evaluating medium-term outcomes between those who receive US, compared with those who do not.

## Supplementary Material

kead546_Supplementary_Data

## Data Availability

The datasets are available from the corresponding author on reasonable request.
