# Supplementary material for: The usefulness of ultrasound in predicting outcomes in patients with shoulder pain: a prospective observational study
Source: Rheumatology (Oxford). 2023 Oct 20;63(8):2162–9. doi: 10.1093/rheumatology/kead546 (PMC11292045; doi:10.1093/rheumatology/kead546)
Supplement: kead546_Supplementary_Data [file kead546_supplementary_data.docx]

**Supplementary methods**

Rasch Model Analysis

For scales that fit the Rasch model, the raw, ordinal scores can be transformed to a linear, interval level scale for parametric analysis. For the Rasch-transformed SPADI, the logit scores were transformed to match the original 0-100 scaling. Where items were found to be locally dependent, with item residual correlations 0.2 or more above the average(1, 2), superitems or ‘testlets’ were created(3). The HADS was found to fit the Rasch model as a total score, indicating that the anxiety and depression subscales could be combined into one over-arching domain of psychological distress. Prior to Rasch analysis the brief illness perception questionnaire (IPQ) (which is intended as a suite of single-indicator health outcomes as opposed to items in a single scale) was investigated using exploratory factor analysis, which revealed that 5 items were loaded by one factor but 3 were loaded by another factor. The three items were reverse-coded with respect to the others which may have been responsible for this apparent multidimensionality. The five items loading onto one factor were assessed for fit to the Rasch model and were found to fit; the Rasch-transformed estimates from these five items are referred to as the brief IPQ in the rest of the analysis.

For all scales, observations were restricted to the baseline visit to assess fit to the model; estimates for subsequent visits were then obtain having anchored the data to the baseline model. Fit to the model was assessed in terms of: item-trait interaction test (acceptable if p>0.05), Cronbach’s alpha (minimum 0.7), individual item fit (acceptable if Bonferroni adjusted item chi-square p>0.05, absolute fit residual <2.5), differential item functioning (acceptable if analysis of variance main effect Bonferroni adjusted p>0.05 for both items for age, sex and shoulder side, or, if p<0.05, effect size for difference in person estimates before and after splitting for DIF <0.2).

Missing Data

Imputation models included each of the individual pathology indicators (coded 0=absent, 1=present), duration of symptoms, patient-reported physiotherapy and number of injections received prior to baseline, using arms to rise from a chair, injection received at time of US scan, EQ5D-5L (Index score and VAS), SPADI, OSS, pain self-efficacy questionnaire (PSEQ), brief IPQ, the Hospital Anxiety and Depression Scale (HADS), the painDetect scale and patient acceptable symptom state (coded unacceptable=0, acceptable=1). Symptom duration, EQ5D index score and EQ5D VAS were imputed using predictive mean matching. Binary variables were imputed using logistic regression. Injection count was imputed using ordinal logistic regression. All remaining variables were imputed using linear regression.

Latent Class Analysis

Additional model fit statistics included AIC, ‘consistent’ AIC (CAIC), adjusted-BIC (a-BIC), entropy, G2 and the proportion of 1000 random seeds associated with the best model – an indicator of model stability.

The number of groups present was determined without covariates in the LCA model. To reduce bias in the analyses following LCA, the final LCA model included covariates. All independent variables and outcomes included in imputation and analysis models were included. This ensured that the association between the latent classes and each of the covariates was maintained(4).

The maximum posterior probability of membership was used to determine the most likely group membership for each patient. For each group the corresponding mean posterior probability was calculated to give an indication of the accuracy of group assignment. This process (LCA and classification) was performed in each imputed dataset independently; group membership was therefore a super-varying variable in subsequent analyses.

Penalised Regression

Least Absolute Shrinkage and Selection Operator (LASSO) regression was used to identify a parsimonious model of total SPADI at six months. This shrinks model coefficients using a shrinkage parameter called lambda. Optimal lambda values were identified via 10-fold cross validation using a starting grid of 100 possible lambda values. The final model was estimated using the largest lambda value that was found to lie within 1 standard error of the value that minimized the mean-squared prediction error.

Growth Mixture Modelling

The function hlme in the R package lcmm(5) was used to identify groups with similar SPADI trajectories over time via growth mixture modelling (GMM). This is similar to latent class growth analysis (LCGA), but allows within-group variation in baseline values (intercepts) and changes over time (slopes) instead of constraining these to be fixed within each group.

Each model included the outcome, total SPADI score, and both week and week^2^ as predictors, to capture non-linear change. Week was centred prior to analysis. No additional covariates were included. To avoid the potential to choose a suboptimal model if there are multiple possible solutions, multiple runs of each model were conducted from a grid of 100 random starting values and the best performing model after 50 iterations was chosen. Successive models with differing numbers of trajectory groups were compared using Bayesian Information Criterion (BIC). Plots of individual trajectories within putative trajectory groups were also examined to ensure consistency, and are provided in the main text for the final model (Figure 1). Posterior probabilities of group membership were used to assign patients to trajectory groups, and mean posterior probabilities in each trajectory group were examined to confirm that patients were classified with adequate accuracy.

Patients were eligible for inclusion in this analysis if they had SPADI data available at 6 months (n=330). Two patients with apparently outlying (extremely low) SPADI values at baseline, which were not data entry errors, caused issues for model convergence and were excluded (final n=228).

**Supplementary results**

Latent Class Analysis

Of the additional fit statistics, AIC, a-BIC & G2 favoured the 4 group solution, while BIC, CAIC & entropy favoured the 3 group solution. The overall proportion of 1000 random seeds associated with the final model, where a high proportion is favourable, did not favour either solution (both 95%) (Table S3).

Growth mixture modelling

A four trajectory solution had the lowest absolute BIC (Table S8). Average posterior probability of group membership was >0.9 in all groups (0.96, 0.98, 0.91 & 0.95 in trajectory groups 1-4 respectively).

**Supplementary Table S1 Ultrasound pathology definitions**

| **Pathology** | **Definition** |
| --- | --- |
| Rotator cuff tears (full or partial) | Record foot print tears as partial thickness if articular surface not involved |
| Bursal thickening | Bursal effusion or synovitis >0.5mm |
| Dynamic subacromial impingement | Bunching of the bursa lateral to the coraco-acromial ligament during active abduction in the absence of rotator cuff tear |
| Calcific tendinitis | Diagnosed in the presence of globular calcific deposition, exclude linear entheseal calcifications |
| Acromioclavicular joint pathology | Diagnosed in the presence of osteophytosis or synovitis, effusion, joint malalignment and bone cortex irregularity |
| Glenohumeral osteoarthritis | Diagnosed by the presence of marginal osteophytosis at the cartilage bone junction or humeral articular cartilage thinning |
| Adhesive capsulitis/ frozen shoulder | Diagnosed in patients with limited passive external rotation possibly with a small long head of biceps effusion and thickening of the coracohumeral ligament compared to the contra-lateral side in the absence of any features of osteoarthritis |
| Biceps tenosynovitis | Non-displaceable or non-fully compressible hypoechoic thickening of the long head of biceps tendon sheath with or without power Doppler signal of evidence of tendinopathy or thinning of the long head of biceps tendon |
| Rotator cuff tendinopathy | Hypoechogenicity or heterogeneity with thickening of the rotator cuff tendons compared to the other rotator cuff tendons of the ipsilateral or contralateral shoulder |

**Supplementary Table S2 Summary of Rasch model solutions**

| **Scale** | **Item-trait Chisq**  **P value** | **Mean (SD) person location, logits** | **CA*** | **Comments** |
| --- | --- | --- | --- | --- |
| SPADI | p=0.896 | 0.09 (0.42) | 0.77 (n=457) | One testlet with low fit residual =-2.67 |
| OSS | p=0.925 | -0.55 (1.40) | 0.91 (n=474) | None |
| painDetect | p=0.148 | -0.62 (0.64) | 0.70 (n=450) | None |
| PSEQ | p=0.990 | 1.21 (1.55) | 0.95 (n=458) | p<0.05 DIF by age; effect size =0.05 |
| HADS | p=0.590 | -1.50 (1.28) | 0.87 (n=451) | p<0.05 DIF by sex; effect size =0.16 |
| Shoulder activity | p=0.244 | -0.30 (0.95) | 0.75 (n=453) | p<0.05 DIF by age; effect size =0.07  p<0.05 DIF by sex; effect size =0.01 |
| Brief IPQ | p=0.456 | 0.06 (0.38) | 0.77 (n=423) | p<0.05 DIF by age; effect size =0.03 |

CA=Cronbach’s alpha; Chisq=Chi-squared; DIF=Differential item functioning; HADS=hospital anxiety and depression scale; IPQ=illness perception questionnaire; P-SEQ=pain self-efficacy questionnaire; SPADI=shoulder pain and disability index

*Excluding patients with missing data and those with ‘extreme’ scores (i.e. at the margins of the distribution)

**Supplementary Table S3 Model fit and stability measures for solutions with 2 to 6 classes**

| **Number of classes** | **df** | **AIC** | **BIC** | **CAIC** | **a-BIC** | **Entropy** | **G^2^** | **Solution %** | **BLRT p-value*** |
| --- | --- | --- | --- | --- | --- | --- | --- | --- | --- |
| 1 | 502 | 471.0 | 508.9 | 517.9 | 480.4 | 1.00 | 453.0 | 100% | - |
| 2 | 492 | 374.6 | 454.7 | 473.7 | 394.4 | 0.74 | 336.6 | 36% | p=0.010 |
| 3 | 482 | 259.1 | 381.3 | 410.3 | 289.2 | 0.89 | 201.1 | 95% | p=0.010 |
| 4 | 472 | 221.3 | 385.7 | 424.7 | 261.9 | 0.76 | 143.3 | 95% | p=0.010 |
| 5 | 462 | 221.9 | 428.4 | 477.4 | 272.9 | 0.79 | 123.9 | 21% | p=0.800 |
| 6 | 452 | 219.2 | 467.8 | 526.8 | 280.5 | 0.80 | 101.2 | 39% | p=0.680 |

AIC=Akaike’s information criterion; (a-)BIC=(sample size adjusted) Bayesian information criterion; BLRT=Bootstrapped likelihood ratio test; CAIC=consistent AIC; df=degrees of freedom; G^2^=likelihood ratio statistic

*Compares fit of model with k classes to that of model with k-1 classes

**Supplementary Table S4: Groups derived via LCA separately in previous retrospective audit (n=3000) and current study (n=500)**

|  | **All patients** | |  | **Groups identified in audit of n=3000 scans*** | | | |  | **Groups identified in the current study** | | | |
| --- | --- | --- | --- | --- | --- | --- | --- | --- | --- | --- | --- | --- |
|  | **n=3000** | **n=500** |  | **Group 1** | **Group 2** | **Group 3** | **Group 4** |  | **Group 1** | **Group 2** | **Group 3** | **Group 4** |
| RC tear, yes/no, % |  |  |  |  |  |  |  |  |  |  |  |  |
| Full-thickness RC tear, % |  |  |  |  |  |  |  |  |  |  |  |  |
| Bursitis, % |  |  |  |  |  |  |  |  |  |  |  |  |
| Impingement, % |  |  |  |  |  |  |  |  |  |  |  |  |
| Calcific tendinitis, % |  |  |  |  |  |  |  |  |  |  |  |  |
| ACJ degeneration, % |  |  |  |  |  |  |  |  |  |  |  |  |
| Glenohumeral OA, % |  |  |  |  |  |  |  |  |  |  |  |  |
| Adhesive capsulitis, % |  |  |  |  |  |  |  |  |  |  |  |  |
| Biceps tenosynovitis, % |  |  |  |  |  |  |  |  |  |  |  |  |
| Rotator cuff tendinopathy, % |  |  |  |  |  |  |  |  |  |  |  |  |

| **0** | **10** | **20** | **30** | **40** | **50** | **60** | **70** | **80** | **90** | **100** |
| --- | --- | --- | --- | --- | --- | --- | --- | --- | --- | --- |

***** see reference (6)

ACJ=acromioclavicular joint; LCA=latent class analysis; OA = osteoarthritis; RC=rotator cuff;

**SupplementaryTable S5: Proportions of patients who received a steroid injection at their ultrasound scan by US-detected pathology**

|  | **Injection received at scan % (n)** | |
| --- | --- | --- |
|  | **Pathology absent** | **Pathology present** |
| RC tear | 49% (114/233) | 15% (15/97) |
| Bursitis | 15% (14/96) | 49% (115/234) |
| Impingement | 38% (46/122) | 40% (83/208) |
| Calcific tendinitis | 39% (118/301) | 38% (11/29) |
| ACJ degeneration | 45% (71/157) | 34% (58/173) |
| Glenohumeral OA | 39% (125/317) | 31% (4/13) |
| Adhesive capsulitis | 41% (123/301) | 21% (6/29) |
| Biceps tenosynovitis | 40% (125/316) | 29% (4/14) |
| Rotator cuff tendinopathy | 38% (85/223) | 41% (44/107) |

**Supplementary Table S6: Estimated probabilities of post baseline treatment by US pathology group**

| **Treatment** | **Model** | **Bursitis ( w/o ACJ degeneration)** | **Bursitis (with ACJ degeneration)** | **RC tear** | **No bursitis or RC tear** | **Overall P value** |
| --- | --- | --- | --- | --- | --- | --- |
| Opioids | Unadjusted | 0.08 (0.02, 0.14) | 0.18 (0.10, 0.26) | 0.13 (0.05, 0.21) | 0.08 (0.01, 0.16) | p=0.197 |
|  | Adjusted* | 0.12 (0.04, 0.20) | 0.18 (0.10, 0.25) | 0.09 (0.03, 0.15) | 0.10 (0.02, 0.18) | p=0.306 |
| NSAIDs | Unadjusted | 0.04 (0.00, 0.08) | 0.14 (0.07, 0.21) | 0.12 (0.04, 0.20) | 0.05 (-0.01, 0.11) | p=0.127 |
|  | Adjusted* | 0.05 (-0.01, 0.11) | 0.14 (0.07, 0.20) | 0.09 (0.02, 0.15) | 0.06 (0.00, 0.13) | p=0.320 |
| Physiotherapy (patient-reported) | Unadjusted | 0.57 (0.46, 0.68) | 0.52 (0.42, 0.63) | 0.65 (0.54, 0.76) | 0.59 (0.45, 0.72) | p=0.476 |
|  | Adjusted* | 0.55 (0.43, 0.66) | 0.53 (0.42, 0.63) | 0.67 (0.56, 0.78) | 0.58 (0.45, 0.71) | p=0.337 |
| Injections (patient-reported) | Unadjusted | 0.60 (0.49, 0.71) | 0.56 (0.44, 0.68) | 0.36 (0.22, 0.49) | 0.38 (0.24, 0.52) | p=0.013 |
|  | Adjusted* | 0.61 (0.49, 0.72) | 0.56 (0.44, 0.68) | 0.35 (0.21, 0.49) | 0.38 (0.24, 0.53) | p=0.018 |
| Surgery (patient-reported**) | Unadjusted | 0.12 (0.05, 0.19) | 0.05 (0.00, 0.10) | 0.25 (0.15, 0.35) | 0.10 (0.02, 0.18) | p=0.006 |
|  | Adjusted* | 0.11 (0.04, 0.18) | 0.05 (0.00, 0.10) | 0.26 (0.15, 0.38) | 0.10 (0.02, 0.18) | p=0.011 |

*Adjusted for age, sex and symptom duration **Imputed surgery=yes for 4 patients confirmed to have had surgery who did not report it

ACJ=acromioclavicular joint; NSAID=non-steroidal anti-inflammatory drug; RC=rotator cuff; w/o=without;

**Supplementary Table S7 Linear regression of total SPADI at 6 months on individual pathologies (imputed data)**

|  | **Coefficient* (95% CI), p-value** | |
| --- | --- | --- |
| **Baseline characteristic** | **Including adhesive capsulitis**  **n=330*** | **Excluding adhesive capsulitis**  **n=301**** |
| Age, years | 0.01 (-0.17, 0.18), p=0.953 | 0.04 (-0.14, 0.22), p=0.653 |
| Female | -1.70 (-5.66, 2.26), p=0.399 | -1.82 (-6.04, 2.39), p=0.396 |
| RC tear | 2.63 (-2.30, 7.57), p=0.295 | 2.84 (-2.39, 8.07), p=0.286 |
| Bursitis | -1.73 (-6.54, 3.07), p=0.478 | -1.86 (-7.07, 3.35), p=0.483 |
| Impingement | -0.27 (-4.74, 4.20), p=0.905 | -0.40 (-5.14, 4.35), p=0.869 |
| Calcific tendinitis | -2.84 (-9.48, 3.79), p=0.400 | -3.48 (-10.60, 3.64), p=0.337 |
| ACJ degeneration | -0.07 (-4.09, 3.95), p=0.973 | -1.14 (-5.49, 3.20), p=0.604 |
| Glenohumeral OA | 5.01 (-4.96, 14.98), p=0.324 | 4.42 (-6.16, 15.01), p=0.411 |
| Adhesive capsulitis | -2.29 (-9.34, 4.75), p=0.522 | - |
| Biceps tenosynovitis | -3.67 (-13.25, 5.91), p=0.452 | -5.20 (-15.67, 5.28), p=0.329 |
| Rotator cuff tendinopathy | 0.76 (-3.52, 5.05), p=0.726 | 0.75 (-3.79, 5.29), p=0.745 |
| Injection at time of scan | 4.21 (-0.21, 8.64), p=0.062 | 4.06 (-0.64, 8.77), p=0.090 |
| Duration of symptoms, months | 0.00 (-0.04, 0.05), p=0.890 | 0.01 (-0.06, 0.09), p=0.710 |
| Uses arms to rise from chair | 2.17 (-2.05, 6.39), p=0.312 | 1.94 (-2.65, 6.53), p=0.405 |
| Had physiotherapy before baseline | 0.07 (-4.53, 4.67), p=0.977 | 0.38 (-4.65, 5.41), p=0.882 |
| Had 1 injection before baseline | 2.31 (-2.98, 7.60), p=0.391 | 2.11 (-3.76, 7.97), p=0.480 |
| Had 2 injections before baseline | 5.96 (-3.23, 15.14), p=0.203 | 6.89 (-2.95, 16.73), p=0.169 |
| Total SPADI score | 0.62 (0.35, 0.90), p<0.001 | 0.58 (0.29, 0.87), p<0.001 |
| Shoulder activity score | -0.59 (-1.16, -0.03), p=0.040 | -0.61 (-1.24, 0.02), p=0.059 |
| P-SEQ | -0.20 (-0.47, 0.07), p=0.141 | -0.20 (-0.49, 0.08), p=0.156 |
| Brief IPQ | 0.17 (-0.19, 0.53), p=0.348 | 0.16 (-0.23, 0.55), p=0.426 |
| HADS | 0.03 (-0.37, 0.43), p=0.888 | 0.02 (-0.41, 0.45), p=0.927 |
| painDetect | -0.01 (-0.42, 0.40), p=0.955 | 0.08 (-0.36, 0.53), p=0.710 |
| Constant | 38.43 (32.02, 44.83), p<0.001 | 39.32 (32.43, 46.20), p<0.001 |
| R^2^; Adjusted R^2^ | 0.28;0.23 | 0.28; 0.22 |

*170 patients did not complete total SPADI at 26 weeks **29 patients had adhesive capsulitis and were excluded; ACJ=acromioclavicular joint; HADS=hospital anxiety and depression scale; IPQ=illness perception questionnaire; P-SEQ=pain self-efficacy questionnaire; RC=rotator cuff; SPADI=shoulder pain and disability index

**Supplementary Table S8:** Bayesian information criteria (BIC) for a number of growth mixture models with differing numbers of trajectories

| **Number of trajectories** | **BIC** |
| --- | --- |
| 1 | 7264.7 |
| 2 | 7166.5 |
| 3 | 7148.3 |
| 4 (final model) | 7091.7 |
| 5 | 7144.9 |

***Supplementary Figure S1 Venn diagram of the major pathologies present in group 4***

*To enable this figure to be drawn, group 4 was defined as patients with no bursitis or RC tear present, rather than using the US groups assigned via multiple imputation for missing covariate data, which differed between imputed datasets.*

*Of the 37 patients without one of the pathologies included here, 34 had no pathology, 2 had ACJ degeneration and 1 had glenohumeral OA.*

**

**Supplementary references**

1. Marais I, Andrich D. Formalizing dimension and response violations of local independence in the unidimensional Rasch model. Journal of applied measurement. 2008;9(3):200-15.

2. Christensen KB, Makransky G, Horton M. Critical Values for Yen’s Q3: Identification of Local Dependence in the Rasch Model Using Residual Correlations. Appl Psychol Meas. 2016;41(3):178-94.

3. Wainer H, Kiely GL. Item Clusters and Computerized Adaptive Testing: A Case for Testlets. Journal of Educational Measurement. 1987;24(3):185-201.

4. Bray BC, Lanza ST, Tan X. Eliminating Bias in Classify-Analyze Approaches for Latent Class Analysis. Struct Equ Modeling. 2015;22(1):1-11.

5. C P-L, V P, B L. Estimation of Extended Mixed Models Using Latent Classes and Latent Processes: The R Package lcmm. Journal of Statistical Software. 2017;78(2):1-56.

6. Tran G, Hensor EM, Ray A, Kingsbury SR, O'Connor P, Conaghan PG. Ultrasound-detected pathologies cluster into groups with different clinical outcomes: data from 3000 community referrals for shoulder pain. Arthritis research & therapy. 2017;19(1):30.
